# Prevalence of complications of male circumcision in Anglophone Africa: a systematic review

**DOI:** 10.1186/1471-2490-7-4

**Published:** 2007-03-02

**Authors:** Adamson S Muula, Hans W Prozesky, Ronald H Mataya, Joseph I Ikechebelu

**Affiliations:** 1 Department of Community Health, University of Malawi, College of Medicine, Blantyre, Malawi; 2 Department of Epidemiology, Denis and Joan Gillings School of Global Public Health, University of North Carolina at Chapel Hill, USA; 3 Department of Medicine, Health Sciences Faculty, University of Stellenbosch, Tygerberg, South Africa; 4 Department of Global Health, School of Public Health, Loma Linda University, California, USA; 5 Department of Obstetrics and Gynaecology, Faculty of Medicine, Mnamdi Azikiwe University, Nnewi Campus, Anambra State, Nigeria

## Abstract

**Background:**

There is growing evidence that male circumcision (MC) prevents heterosexual acquisition of HIV by males in sub-Saharan Africa, the region of the world heavily affected by the HIV pandemic. While there is growing support for wide-spread availability and accessibility of MC in Africa, there is limited discussion about the prevalence of physical complications of male circumcision on the continent.

**Methods:**

A systematic literature search and review of articles in indexed journals and conference abstracts was conducted to collect and analyze prevalence of complications of MC in Anglophone sub-Saharan Africa. Information extracted included: indications for MC, complications reported, age of patients and category of circumcisers.

**Results:**

There were 8 articles and 2 abstracts that were suitable for the analysis. The studies were not strictly comparable as some reported on a wide range of complications while others reported just a limited list of possible complications. Prevalence of reported complications of MC ranged from 0% to 50.1%. Excluding the study with 50.1%, which was on a series of haemophilia patients, the next highest prevalence of complications was 24.1%. Most of the complications were minor. There was no firm evidence to suggest that MCs performed by physician surgeons were associated with lower prevalence of complications when compared with non-physician health professionals.

**Conclusion:**

The available data are inadequate to obtain a reasonable assessment of the prevalence of complications of MC in sub-Saharan Africa. Some of the available studies however report potentially significant prevalence of complications, though of minor clinical significance. This should be considered as public health policy makers consider whether to scale-up MC as an HIV preventative measure. Decision for the scale-up will depend on a careful cost-benefit assessment of which physical complications are certainly an important aspect. There is need for standardized reporting of complications of male circumcision.

## Background

The HIV pandemic is among the most critical public health challenges facing the African continent. Observational studies and three randomized controlled trials (RCTs) have reported that male circumcision can prevent HIV acquisition heterosexually by men [1-3]. Williams et al [4] have conducted mathematical models showing that wide-scale implementation of MC has the potential of reducing 2.0 million (1.1–3.8 million) new HIV infections in the first ten years of implementation in sub-Saharan Africa.

Several studies have reported on the acceptability of male circumcision for the prevention of HIV in sub-Saharan Africa [5-7]. In a review, Westercamp and Bailey report that there is sufficient research evidence to suggest that male circumcision is acceptable in sub-Saharan Africa for the prevention of HIV transmission [8]. However, Mills and Siegfried have suggested a "cautious optimism" approach towards male circumcision [9]. There is on-going debate as to whether male circumcision should be routinely performed for the purpose of preventing heterosexual transmission of HIV in sub-Saharan Africa [10-15].

While the literature has focused on the role of MC in the prevention of transmission of HIV in sub-Saharan Africa, we argue that there has not been commensurate interest in the estimation of safety and complications potentially associated with any scale-up in MC in Africa. In conducting this review, we were motivated to find out the available evidence regarding the reported prevalence of complications of MC in Africa. We therefore conducted a review of the published literature to attempt to answer the question: What has been the reported prevalence of complications of MC in sub-Saharan Africa? A secondary question is: Who is conducting MC in Africa?

## Methods

### Search strategy and selection criteria

We searched for all reports in peer-reviewed journals that reported on complications of male circumcision in sub-Saharan Africa and abstracts from the International Conference on AIDS (2004 and 2006). Journal databases searched included: African Journals Online (AJOL), MEDLINE, OVID, Google Scholar and Web of Science. The search period was limited to 1980 and August 2006. We used the key word: "circumcision". Studies were included for analysis if they reported on any series of patients in Anglophone Africa on whom male circumcision had been conducted and complications reported. ASM, RM and JII participated in the data collection. Data abstracted included: author details, location of study, number of patients circumcised in the reported series, ages of patients, inclusion criteria for procedure, prevalence of complications, nature of complications reported, period of follow-up for reporting of complications, indications for circumcision and who performed the circumcisions. Only studies published in English were included, irrespective of study design. Our method used applicable recommendations from the Meta-Analysis of Observational Studies in Epidemiology (MOOSE) guidelines as reported by Stroup et al [16].

## Results

### Clinical characteristics of cases

Eight articles and two conference abstracts were considered suitable for the review. The papers reported on studies conducted in Kenya, Nigeria, South Africa and Tanzania. Table 1 reports on indications reported for MC, exclusion criteria of cases, length of period of follow-up and the range of complications reported, which included zero reporting. Zero reporting means type of complication was reported as having never occurred.

Studies included randomized controlled clinical trials and reports of routine clinical work. The inclusion and exclusion criteria in the series of cases were diverse with the RCTs reporting stringent inclusion criteria of cases while the routine series of patients were generally less restrictive. The lists of complications reported in studies were different. The period of follow-up to document complications were variable ranging from just immediate post-operative up to a year. 4 of the 10 studies did not report follow-up time.

### Complications of male circumcision

Table 2 reports on the total number of cases in a series group, age of patients, categories of circumciser and prevalence of complications reported. The prevalence of complication reported ranged from 0% to 50.1% in a series of hemophiliacs. Different professional categories performed circumcision. There were equivocal reports on the comparison of complications between circumcisions performed by nurses versus those performed by physicians.

### Association between category of circumciser and complications

Several studies reported high prevalence of complications when circumcision was conducted by untrained personnel. Osuigwe et al [23] reported that midwives were associated with 30.6% complications compared to physicians at 14.5%. However, physicians at a university teaching hospital were associated with fewer complications compared to private and mission hospital physicians. The series of cases by Magoha [26] and Manji [25] were operated on by one physician in each case and no comparisons could be made about the skill of the circumciser. Auvert et al study [20] used general physicians for all their subjects. Okeke et al [19] reported more complications with nurses compared to physicians. However, the difference was not statistically significant.

## Discussion

There is limited published literature on the complications of male circumcision in sub-Saharan Africa. The available evidence regarding the prevalence of complications in male circumcision is conflicting, with some studies reporting significantly high complications prevalence and at least one study reporting no complications. It is plausible to consider that a study that may not have reported a single complication may indeed have none to report or that complications may have been consider so minor as to be 'worth' reporting. In case of complications that may be experienced at home and there are significant barriers to access medical care, it is conceivable to expect that many patients will not present to health facilities for care following minor to moderate complications. Another explanation for no report of complications may be that the patients may have presented for management of complications at a different facility from where they obtained circumcision.

In virtually all the studies reviewed, the majority of complications reported were of minor clinical consequence. The exception was the study by Shittu and Shokunbi [24] which reported on a special group of patients i.e. hemophiliacs, with extremely high risk of bleeding from minimal trauma. The interpretation of prevalence of complications in this study must be made within these special circumstances. Other than the report of bleeding, no other complication was reported. It is unlikely though that bleeding was the only complication experienced; rather it would seem that this was the main focus of the study.

Reports on complications of male circumcision also differ in so far as the time after the circumcision that complications were reported i.e. early versus late complications. Yegane et al [27] for instance have reported on possible late complications of MC which include granuloma formation, penile rotation and secondary chordee [27]. In some of the studies that were reviewed in our study like that of Bailey et al [17] where patients were followed up for complications for 8 days, long term complications such as keloid formation may have been missed. Bailey et al's randomized controlled study however was of much longer duration and the assessment of long term complications was possible, but not reported. In general, most of the studies we reviewed reported on bleeding and infections only. Auvert's et al study however had reported an extensive list of possible complications and included zero reporting. Of the complications reported by Auvert et al, 13 (31.7%) reported pain, excessive bleeding (15%), infection (5%), damage to penis (5%), 1 anesthetic complications and 9 complaints of problems with appearance.

The prevalence of complications of MC in North America has been reported in the range of 0.2% to 0.5% [28]. However, the true prevalence of complications is not known as ways of ascertaining these will always underestimate the true prevalence [28]. Ben Chaim et al [29] have reported on series of 19,478 male infant circumcision conducted in Israel, of which only 66 (1.3%) recorded complications. This study reported fewer complications than most of the studies we reviewed but prevalence was higher than the single study that reported no complications.

The study by Okafor et al [22] which reported no complications, had potentially 119 neonates but 17 were excluded for a variety of reasons. This could suggest that proper selected of MC candidates will be necessary to minimize complications. However, such conclusion may not be justified in the absence of data on the criteria for exclusion i.e. the selection may have or may have not influenced the prevalence of complications.

There are always concerns about the safety of MC in sub-Saharan African health systems. Mattson et al reported on the feasibility of performing MC in health facilities in Kenya. The lack of basic instruments and inadequate supplies were identified as crucial limitations in facilitating provision of safe MC in that country [30]. Ahmed [31] studied circumcision practices in Anjouan, Comoros and reported that circumcisions by trained lay people were as safe as those carried out by physicians in hospitals. Training and supervision was emphasized in this series.

The special cases of complications in haemophiliacs deserve special mention. It has been reported that the use of fibrin glue or the "diathermic knife" may prevent bleeding and transfusion with blood factors [32,33]. These supplies will need to be considered if MC is to be scaled-up, but will increase the cost of the procedure.

It is reasonable to expect that prevalence of complications from MC in Africa is likely to be higher than in countries with well-resourced health systems. While that expectation is reasonable and perhaps will be realized, a recent publication by Archibald et al is also informative [34]. Archibald et al compared contamination of blood culture specimens at Duke Medical Center (Durham, North Carolina, United States), Kamuzu Central Hospital (Malawi) and Muhimbili National Hospital (Tanzania). Contamination of specimens at both African centers was less than 2% while it was 28% at Duke Medical Center. The expectation that developing country health systems will always perform poorly when compared to developed nation health systems may therefore not be justified.

## Conclusion

The literature on prevalence of complications following male circumcision in sub-Saharan Africa is limited. Most of the complications reported have been of minor clinical consequence. Reporting practices also have been varied such that comparisons among studies with regard to age of patients, indications for circumcision, duration of follow-up of complications and categories of circumcisers are difficult. There is need to develop and encourage standardized reporting of complications of male circumcisions. We believe that the finding that there is not enough data to suggest that MC is associated with more than the 'average' risk in Africa is important as public policy discussions gain momentum as whether to scale-up provision and accessibility of the procedure to prevent HIV transmission.

## Competing interests

The author(s) declare that they have no competing interests.

## Authors' contributions

ASM conceived the idea of the study, participated in identifying papers for review and drafting of manuscript. ASM is guarantor of manuscript.

HWP participated in identifying papers for review, analysis and drafting of manuscript. RM participated in identifying papers for review and drafting of manuscript.

JII participated in identifying papers for review, analysis and drafting of manuscript. All authors approved the manuscript submitted.

## Pre-publication history

The pre-publication history for this paper can be accessed here:



## Figures and Tables

**Table 1:** Socio-clinical characteristics of male circumcision cases included in the review

**Author(s)**	**Reasons for circumcision**	**Exclusion Criteria**	**Period of follow-up**	**Complications reported**
Bailey et al [17]	Randomized controlled trial	HIV infection	8 days	bleeding, infection
Kiwanuka et al [18]	Randomized controlled trial	Not reported	6 weeks	Not reported
Okeke at al [19]	Non medical reasons	Not reported	Not reported	redundant foreskin, excessive skin, skin bridges, amputation of glans, buried penis, hemorrhage
Auvert et al [20]	Randomized controlled trial	Any contraindication to MC and HIV infection	1 month	pain, excessive bleeding, infection, damage to penis, anesthetic complications, excessive skin removal, insufficient skin removal, delayed healing, cosmetic concerns, problems with urination
Krieger et al [21]	Randomized controlled trial	Any medical contraindications to MC e.g. paraphimosis, Significant phimosis, recurrent balanitis, history of bleeding, keloid formation	30 days	infections, bleeding, delayed healing, disrupted wound, swelling, anesthetic, erectile dysfunction
Okafor et al [22]	Feasibility study of MC	Lack of parental consent, preterm birth, congenital anomalies, low APGAR score, history of neonatal jaundice in a sibling, jaundice at birth	1 year	None
Osuigwe et al [23]	Parental request	None reported	6 weeks	bleeding, incomplete circumcision, meatal stenosis, urethral laceration
Shittu and Shokunbi [24]	socio-cultural	None	not reported	bleeding
Manji [25]	socio-cultural, religious	none	not specified	infection, bleeding, haematoma
Magoha [26]	cultural, religious, phimosis, paraphimosis, urinary infection, acute infection, hygienic reasons, enhanced sexual sensation, preputial cyst	None reported	Not reported	wound infection, hemorrhage, retention of urine, penile edema, haematoma, scrotal laceration, wound dehiscence, glans injury, meatal stenosis

**Table 2:** Prevalence of Complications of Male Circumcision Reported From sub-Saharan Africa

**Author**	**Location**	**Total number of patients**	**Ages of Patients**	**Circumcisers**	**Number of complications**	**Prevalence of Complications**
Bailey et al [17]	Kisumu, Kenya	1334	18–24 yrs	Not indicated	23	1.7%
Kiwanuka et [18]	Rakai, Uganda	136	15–49 years	Not indicated	5	3.7%
Okeke et al [19]	Ibadan, Nigeria	322	< 1 yr	Nurses, physicians and traditional circumcisers	65	20.2%
Auvert et al [20]	Orange Farm, South Africa	1568	18–24 yrs	Generalist physicians	60	3.8%
Krieger et al [21]	Kisumu, Kenya	479	18–24 yrs	Physicians and clinical officers	17	3.5%
Okafor et al [22]	Nigeria	102	< 1 yr	Physicians	0	0
Osuigwe et al [23]	Nigeria	138	neonates	Physicians and nurses	33	24.1%
Shittu and Shokunbi [24]]	Ibadan, Nigeria	70 hemophiliacs	birth to 35 yrs	Nurses, physicians, community health workers and traditional circumcisers	35	50.1%
Manji [25]]	Tanzania	386	< 1 yr	Pediatricians	11	2.8%
Magoha [26]]	Lagos, Nigeria and Nairobi, Kenya	249	birth to 54 years	Specialist Surgeon physicians	28	11.2%
